# Supplementary material for: Comparison of Two RNA Extraction Methods for the Molecular Detection of SARS-CoV-2 from Nasopharyngeal Swab Samples
Source: Diagnostics (Basel). 2022 Jun 27;12(7):1561. doi: 10.3390/diagnostics12071561 (PMC9317615; doi:10.3390/diagnostics12071561)
Supplement: Supplementary file 1 [file diagnostics-12-01561-s001.zip › Supplementary Figure S1.pdf]

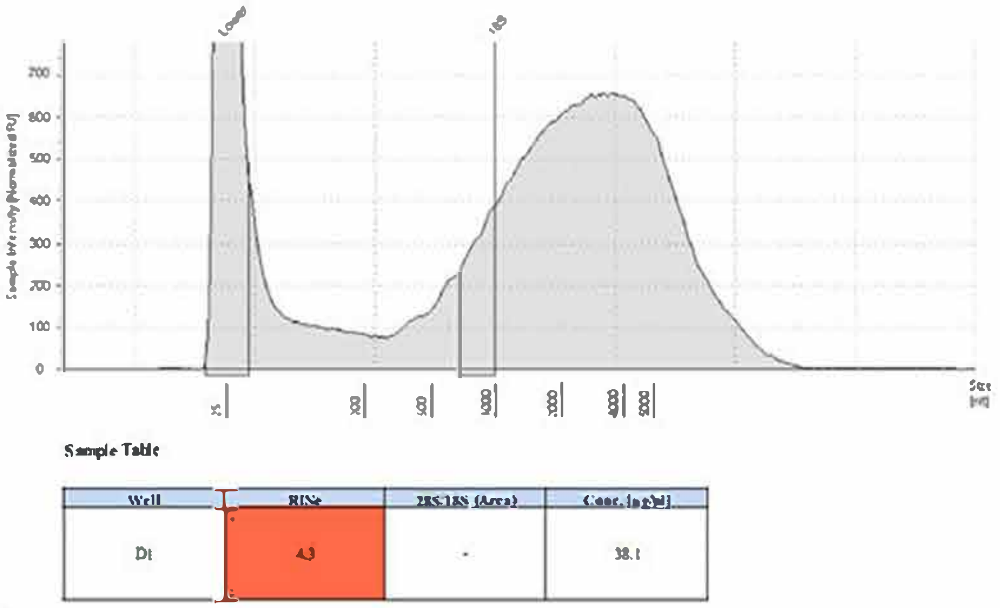

A.

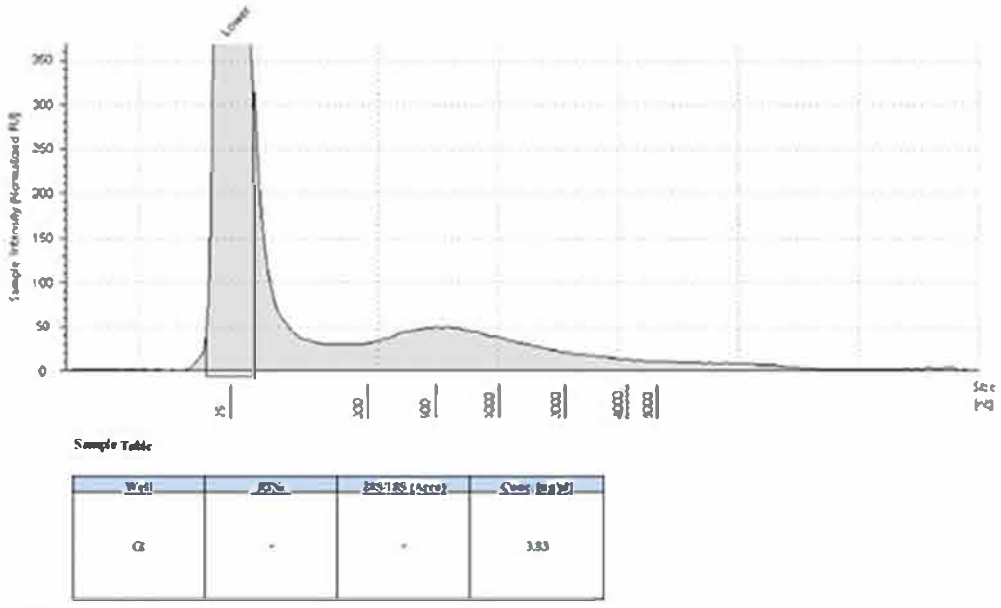

B.

samples	used kit	Nucleic Acid(ng/uL)	A260/A280	A260/A230
D1	kit fast	36.762	1.254	0,055

C.

samples	used kit	Nucleic Acid(ng/uL)	A260/A280	A260/A230
G1	Kit fast	4.92	0.95	0.04

D.

Supplementary Figure S1. Comparison between TapeStation and Nanodrop quantification. Figure S1A and S1B showed elettroferogram and quantification table of two samples extracted by rapid kit. Figure S1C and S1D showed the respective quantification by Nanodrop instrument.
